# A Base-Independent Repair Mechanism for DNA Glycosylase—No Discrimination Within the Active Site

**DOI:** 10.1038/srep10369

**Published:** 2015-05-27

**Authors:** Iris D. Blank, Keyarash Sadeghian, Christian Ochsenfeld

**Affiliations:** 1 Chair of Theoretical Chemistry, Department of Chemistry, University of Munich (LMU), Butenandtstr. 7, D-81377 Munich, Germany; 2 Center for Integrated Protein Science Munich (CIPSM) at the Department of Chemistry, University of Munich (LMU), Butenandtstr. 5-13, D-81377 Munich, Germany

## Abstract

The ubiquitous occurrence of DNA damages renders its repair machinery a crucial requirement for the genomic stability and the survival of living organisms. Deficiencies in DNA repair can lead to carcinogenesis, Alzheimer, or Diabetes II, where increased amounts of oxidized DNA bases have been found in patients. Despite the highest mutation frequency among oxidized DNA bases, the base-excision repair process of oxidized and ring-opened guanine, FapydG (2,6-diamino-4-hydroxy-5-formamidopyrimidine), remained unclear since it is difficult to study experimentally. We use newly-developed linear-scaling quantum-chemical methods (QM) allowing us to include up to 700 QM-atoms and achieving size convergence. Instead of the widely assumed base-protonated pathway we find a ribose-protonated repair mechanism which explains experimental observations and shows strong evidence for a base-independent repair process. Our results also imply that discrimination must occur during recognition, prior to the binding within the active site.

DNA is constantly damaged by radiation, mutagenic chemicals, and reactive oxygen species, which leads to alkylation, hydrolysis, or oxidation of DNA bases. Therefore, the ability of cells to cope with DNA damages is crucial for their survival. Without effective repair machinery these damages can accumulate and soon affect the genomic stability, since they show a higher tendency for mismatches during replication. An increased level of oxidized DNA bases has been found in patients suffering from diseases such as Alzheimer^1^, Parkinson^2^, Multiple Sclerosis^3^, and Diabetes II^4^. Despite this importance, the reaction mechanism and, in particular, the discrimination within the repair process remained unclear, which is the focus of our present work.

We unravel the molecular repair mechanism for the case of the oxidative damage, FapydG (2,6-diamino-4-hydroxy-5-formamido-pyrimidine), (Fig. 1) that has the highest mutation frequency^5^ of oxidative damages. The mechanism has also important implications for discrimination between damaged and undamaged bases. FapydG is repaired by the base excision repair enzyme Fpg (Formamidopyrimidine-DNA glycosylase, also known as MutM)^6^. It is assumed that Fpg slides along the DNA until it recognizes a damage, which is then flipped into the active site. For Fpg a Schiff base intermediate between the excision of the base and the ribose has been found [PDB-code: 1L1Z^7^], revealing bifunctionality (glycosylase and AP lyase activity)^6^. This means, that Fpg excises the base and the ribose successively. Since the base is excised first, base-protonation has been discussed as initial step, however, no strong evidence for such a process was provided sofar. Once the damaged nucleotide has been excised, the resulting gap is going to be filled with the correct nucleotide by additional enzymes^8^,^9^.

In order to provide reliable insights into the repair of FapydG, computational studies are expected to be helpful, since direct experimental insights are highly difficult to obtain. There is only one crystal structure of the trapped educt state available [PDB-code: 1XC8]^10^. So far the proposed mechanism is only based on assumptions and no alternatives to a base-activated process have been explored. In this work, we employ theoretical methods, including quantum-chemical methods within a QM/MM approach, starting from the X-ray structure^10^ in order to illuminate the overall cleavage reaction of FapyG. Since earlier studies have shown that often large QM spheres are necessary for a reliable theoretical description^11^,^12^,^13^,^14^,^15^, we converged the QM size using up to 700 QM atoms with linear-scaling SCF methods^16^,^17^,^18^,^19^.

## Methods

The crystal structure of wild-type Fpg in complex with a double strand DNA-fragment containing carbocyclic FapydG (cFapydG) from *Lactococcus lactis* was used as the starting structure [PDB code: 1XC8]. XLEAP (AmberTool)^20^ has been used to add hydrogen atoms to the X-ray structure, to neutralize the system with sodium ions and to solvate it in a box of explicit TIP3P water^21^ with a buffer of 10 Å around the solute. The parameters for the neutral proline residue and FapydG were taken from Perlow-Poehnelt *et al.*^22^ and Song *et al.*^23^, respectively. We used ANTECHAMBER^24^,^25^ to parametrize cFapydG. For force field molecular dynamics (FF-MD) simulations we used the NAMD engine^26^ with Amber10 force field parameters^20^. Periodic boundary conditions and particle mesh Ewald summation (PME) with a cutoff value of 10 Å were employed (see SI-6.1). For QM/MM structure optimizations the DL-POLY implementation within ChemShell^27^ (AMBER-FF) was combined with density functional theory (DFT) at the BP86-D3/6-31G**^28^,^29^,^30^,^31^,^32^ level of theory (unless specified otherwise) employing the Q-Chem program package^33^ for the QM part. BP86-D3 was chosen for optimization due to its particular low weighted total mean absolute derivation for reaction energies (3.5 kcal/mol)^34^ and its relatively low computational cost. The repair mechanism was calculated using both the adiabatic mapping approach and the nudged elastic band method of the DL-FIND^35^ module implemented in ChemShell^27^ (for system sizes see SI-6.2). The QM region was successively increased up to 700 atoms (see SI-5.1 for QM and relaxed regions). In SI-1 the influence of basis set and DFT-functional variation is shown.

## Results and Discussion

Starting point for our quantum-chemical study of the repair mechanism is the X-ray structure of Fpg in complex with cFapydG. It is important to note that the X-ray structures available for Fpg in complex with DNA not only differ in the damaged base and in the modifications necessary for trapping the educt state in the experiment, but also in the presence of a water molecule in the active site^10^,^36^,^37^. Therefore these influences together with the possible protonation states (not available from X-ray) are first systematically discussed in the following in order to obtain a realistic starting point for simulating the complex repair process: we start with the crystal structure of Fpg containing cFapydG in complex with DNA [PDB-code: 1XC8]^10^, discuss the influence of the carbon analogue, the proper protonation state, and then turn towards elucidating the reaction mechanism.

### Influence of the carbon analogue

**Figure 2:**
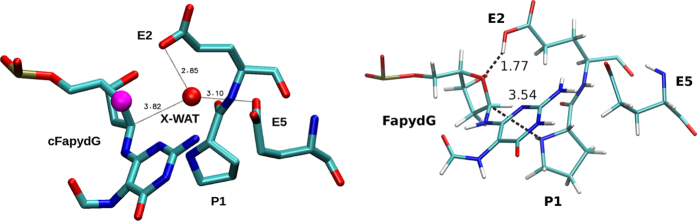
Left: Active site of the X-ray structure of Fpg in complex with cFapydG [PDB code: 1XC8] showing distances for water stabilization. The atomic position of the O 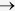 C substitution, which enabled this structure, is highlighted in magenta. Right: Protonation state of the active site of Fpg without X-WAT. E2 is in a protonated form, whereas E5 is not protonated; P1 is neutral.

**Figure 3:**
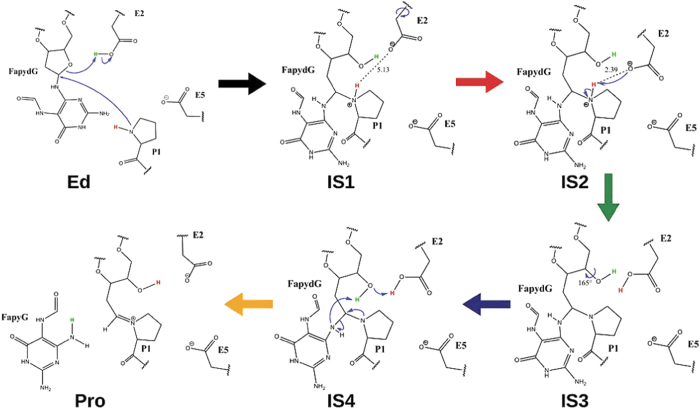
FapydG repair mechanism by Fpg. The color code of the arrows corresponds to the barriers in Fig. 4.

**Figure 4:**
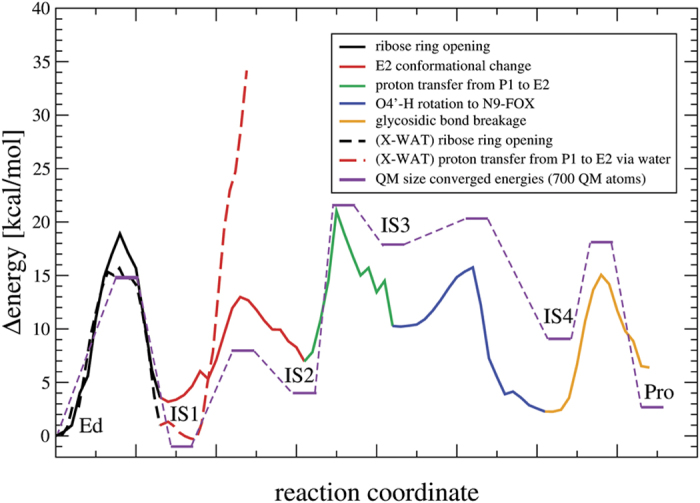
Reaction profile of the repair mechanism of FapydG with the color code of Fig.3. In dashed lines the reaction profile including X-WAT is shown. The system consists of 54412 atoms in total. 10 Å around N_9_ of FapydG are optimized, including 87 QM atoms. The QM size converged energies for the barriers and intermediates using 700 QM atoms are shown in purple.

The only X-ray structure of FapydG^10^ [PDB-Code: 1XC8] shows the educt state, where cFapydG is turned out of the DNA and placed into the active site of Fpg (Fig. 2). To allow crystallization of this reactive state, O_4′_ of the ribose in FapydG has been substituted by a carbon atom, which is a strong hint, that the interaction between O_4′_ and the active site is crucial for the reaction *in vivo*. Within the active site of this structure, a water molecule (X-WAT) has been observed next to the modified 4’-position. For the oxidative guanine damage 8OG (7,8-dihydro-8-oxoguanine), there are X-ray structures available with and without a carbon analogue^36^,^37^ (see SI-2). These structures also differ in the presence of the water molecule. To analyze this difference, we investigate the behavior of the water molecule. We performed FF-MD simulations for the systems containing FapydG with/without X-WAT and cFapydG with/without X-WAT (SI-3.1). In both systems, FapydG and cFapydG with X-WAT, respectively, the presence of X-WAT destabilizes the active site (RMSD plots see SI-3.2) and it is very likely that X-WAT moves out into the solvent. In the combination FapydG with X-WAT, interaction between O_4′_ and the protonated E2 of Fpg cannot be observed. In contrast, the system without X-WAT shows multiple events of E2-O_4′_ interaction (SI-3.3). This interaction is crucial for our proposed base-independent mechanism (see Fig. 3). If it is missing, the mechanism leads to a dead end (see Fig. 4) and can explain, why the carbon analogue allows the crystallization of this reactive state.

Overall, we conclude that due to the substitution of O_4′_ to a C-atom in the X-ray structure^10^, cFapydG is less polar and H-bonds are formed with X-WAT instead of cFapydG. We suggest that the water molecule in the active site is an artifact of the carbocyclic compound cFapydG or c8OG in the X-ray structures [PDB-code: 1XC8^10^ and 4CIS 37], respectively, and is not part of the active site *in vivo*. Therefore, we will not consider the water molecule in the calculations any further.

### Protonation state

The correct protonation state of the active site is clearly decisive for the reaction mechanism. While X-ray data does not provide this information, in principle P1, E2, and E5 (see Fig. 2) can be protonated: However, protonation of the N-terminal P1 can be excluded since no nucleophilic attack at C_1′_ could occur and, consequently, no Schiff base intermediate would be reached. For the two other possibilities, our QM/MM calculations indicate that E2 protonation is favored by 32 kcal/mol over E5 protonation. This is in line with PROPKA^38^,^39^,^40^,^41^ predictions that estimate the pKa of E2 as 7.6 and of E5 as 5.5. This is also in line with the fact that E2 is located closer to the ribose ring than E5, so that most likely the protonated E2 is the proton donor for the first reaction step. The active site for our calculations is shown in Fig. 2.

### Repair mechanism

For Fpg in general, a direct glycosidic bond cleavage mechanism has been proposed for 8OG for many years^42^. Here, the damaged base would be cleaved under nucleophilic attack of P1 while the ribose ring remains intact. Such a direct base excision requires, that the damaged base becomes a better leaving group by protonation. However, for FapydG this seems not possible, since according to our calculations neither energetically favored protonation sites of FapyG exist, nor are there any suitable proton donors in the cavity (as described further below). Furthermore, our QM/MM calculations show, that independent of the protonation state of the active site, the reaction barriers for glycosidic bond cleavage are higher than 30 kcal/mol. In this way, such a mechanism is most unlikely under physiological conditions - independent of the presence of X-WAT (see SI-4.1).

**Figure 5:**
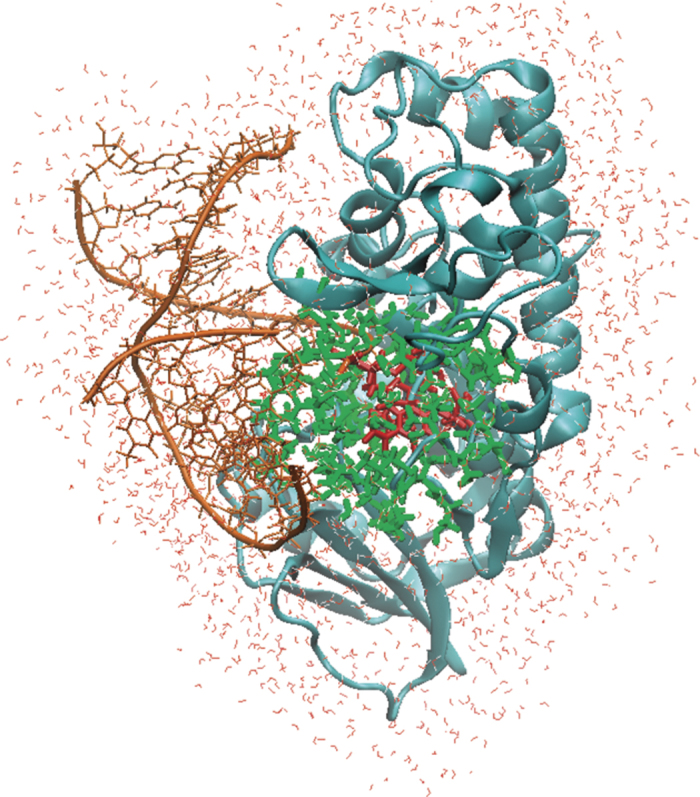
DNA repair enzyme Fpg in complex with damaged DNA. The active site is shown in red, the QM region including 700 atoms is shown in green.

In addition to the direct base-excision pathway, another mechanism has been proposed in the literature for the repair by Fpg, which has received only little attention and for which no evidence has been provided^8^. Here, first the ribose is protonated before excision of the damaged base occurs. This is in line with a recent ribose-protonated mechanism we found for 8OG repair^37^, which, however, is not base-independent. In the first reaction step E2 is deprotonated by O_4′_ while P1 nucleophilic attacks C_1′_ during ribose ring opening leading to IS1 (intermediate state 1; Fig. 3). For this step we calculate a barrier of 14 kcal/mol (see Fig. 4; all energetics listed here are for the converged QM region with 700 atoms; see Fig. 5, SI-5.2 and Section “Details for QM size convergence”). The second reaction step is a reorientation of the E2 side chain (IS2), which allows deprotonation of P1. (As discussed earlier, we have shown X-WAT not to be present in the active site. In case of presence of X-WAT, the first step of the mechanism does not change significantly, while in the second step its presence prevents reorientation of E2, rendering the deprotonation of P1 highly unfavorable. The transfer of the acidic proton of P1 to other residues is due to distance and energetics not accessable under enzymatic conditions. Even the transfer via X-WAT to another residue is energetically unlikely.) In the third step P1 is deprotonated by E2 with a barrier of 17 kcal/mol (IS3). After this proton transfer, the fourth step is the reorientation of the alcohol group at C_4′_ towards the damaged base (IS4) to avoid clashes with the protonated E2 residue. This step was calculated with a barrier of only 3 kcal/mol. The obtained stable intermediate is 8 kcal/mol higher in energy than the initial educt state (Ed). The last of the 5 steps is the base-excision, in which N_9_ is protonated by the alcohol group at C_4′_ which in turn abstracts a proton of E2 (Pro). The glycosidic bond breakes during Schiff base formation between C_1′_ and P1. This crucial reaction step can now occur with a barrier of only 9 kcal/mol. The final product of the cleavage reaction are the free base FapyG and a stable Schiff base (Imine) between the DNA backbone and the N-terminal proline (P1) of Fpg. This product structure is only 2 kcal/mol higher in energy as compared to the initial educt. The obtained Schiff base is also in agreement with the X-ray structure 1L1Z (see SI-4.2). The full repair mechanism is illustrated in Fig.3.

Overall, our repair mechanism is base-independent and can now explain the experimental observations, that a considerable number of different chemically modified DNA bases (pyrimidine^43^,^44^,^45^ and purine bases^10^,^46^) - even nonpolar analogues^47^ - can be excised by Fpg. Despite the structural differences, all these substrates have a N-glycosidic bond. This nitrogen is the only atom of the DNA base that is crucial in the mechanism, since it needs to be protonated to become a neutral leaving group, and is therefore an unspecific target for protonation. This implies, that discrimination of the DNA bases must occur in an earlier step of the DNA-enzyme interaction (recognition).

### Details of QM size convergence

**Figure 6:**
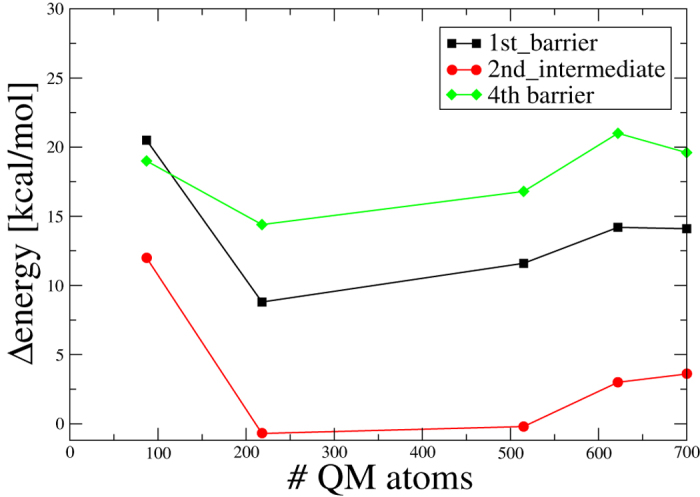
QM size convergence shown for selected points within the repair mechanism.

QM/MM approaches have been widely employed for describing, e.g., complex reactions in enzyme cavities (see, e.g., Ref. 48 for a recent review). Only with advent of linear-scaling QM/MM approaches (e.g., Ref. 19 for a recent review), the full convergence of results with the QM sphere has become possible, where it has been recognized that fairly large QM spheres are necessary for a reliable description of molecular processes^11^,^12^,^13^,^14^,^15^. For the present system, we have performed QM/MM convergence studies with up to 700 QM atoms (see Table 1 and Fig. 6): These indicate that although the reaction profile seems almost converged for 515 QM atoms, relaxation energies upon geometry optimization are only converged for larger spheres with about 700 QM atoms (for details see also SI-5.3). A similar QM size convergence has been found for calculating interaction energies^37^.

## Conclusion

We have presented a new base excision repair mechanism of the oxidative DNA damage FapydG that is base-independent and implies that no discrimination between damaged and undamaged bases occurs within the active site. Instead of the previously assumed direct glycosidic bond cleavage, our calculations strongly suggest a protonation of O_4′_ with ribose ring opening as the first reaction step.

Here, it is important to note that a water molecule within the active site of the X-ray structure is most likely an artifact of employing a carbocyclic analogue to capture the educt state. The observed ribose ring opening as the initial step and the formation of a Schiff base intermediate are also in line with the repair mechanism of 8OG^37^. In difference to 8OG, the opened imidazole ring of FapydG leads to an *anti*-conformation within the active site, a base-unspecific protonation and therefore to a base-independent mechanism. In this way, other oxidative DNA damages, like FapydA^46^, 5-hydroxyuracil^43^ and thymine glycol^44^, can also be excised by Fpg. Even nonpolar analogues of 8OG are excised by Fpg, which has been reported by David and coworkers^47^ and can now be rationalized by our new base-independent mechanism. Overall, we conclude as a consequence of the base-independent mechanism in the enzymatic cavity that discrimination is only part of the base-flip and recognition procedure. We are convinced that our new mechanism will help to elucidate similar DNA repair processes also in other organisms.

## Associated content

The figures were created using VMD^49^. For further details see Supplementary Information.

## Additional Information

**How to cite this article**: Blank, I. D. *et al*. A Base-Independent Repair Mechanism for DNA Glycosylase—No Discrimination Within the Active Site. *Sci. Rep.* **5**, 10369; doi: 10.1038/srep10369 (2015).

## Supplementary Material

Supplementary Information

## Figures and Tables

**Figure 1:**
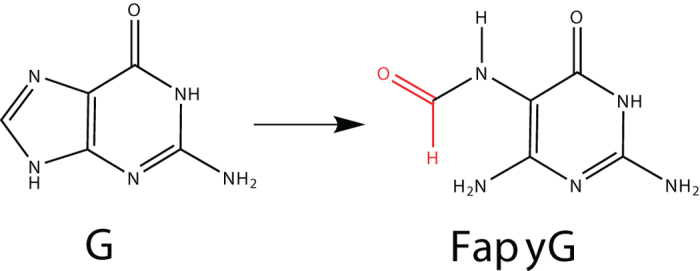
Oxidation of guanine to FapyG (2,6-diamino-4-hydroxy-5-formamido-pyrimidine). We distinguish FapydG as the nucleotide and FapyG as the damaged base.

**Table 1:** Influence of increasing QM region and geometry optimization on the active site.

**size (QM region)**	**max. change in the reaction profile relative to next smaller QM size**	**max. change in the reaction profile due to optimization**	**average relaxation energy (absolute energy)**
218	16.2	7.5	86.9
515	5.5	3.6	24.8
622	4.4	0.9	4.9
700	1.4	1.3	1.1

Energy values in kcal/mol.
