# Supplementary material for: A Base-Independent Repair Mechanism for DNA Glycosylase—No Discrimination Within the Active Site
Source: Sci Rep. 2015 May 27;5:10369. doi: 10.1038/srep10369 (PMC4445063; doi:10.1038/srep10369)
Supplement: Supplementary Information [file srep10369-s1.pdf]

Supplementary Information

A Base-Independent Repair Mechanism for DNA Glycosylase — No Discrimination Within the Active Site

Iris D. Blank[a,b], Keyarash Sadeghian[a,b], and Christian Ochsenfeld[a,b]

[a] Chair of Theoretical Chemistry, Department of Chemistry, University of Munich (LMU), Butenandtstr. 7, D-81377 Munich, Germany

[b] Center for Integrated Protein Science Munich (CIPSM) at the Department of Chemistry, University of Munich (LMU), Butenandtstr. 5-13, D-81377 Munich, Germany

SI-1 Influence of basis set and DFT functional

Fig. SI-1 and Fig. SI-2 show that the deviation between the tested different basis sets and functionals is within 3kcal/mol.

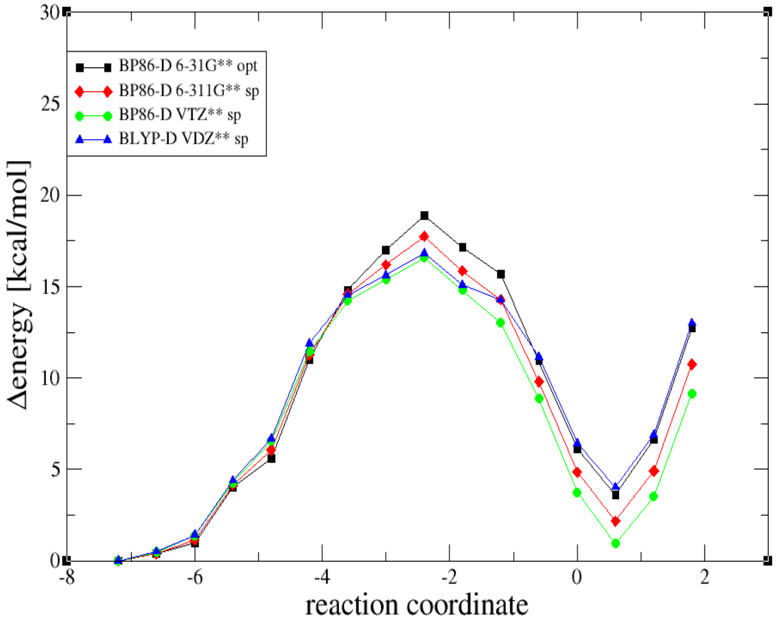

Fig. SI-1: Influence of the basis set and DFT functional on the first reaction barrier, the ribose protonation. All values are relative energies compared to their educt state (sp=single point; opt=optimization). The system consists of 54412 atoms in total and 87 atoms in the QM region.

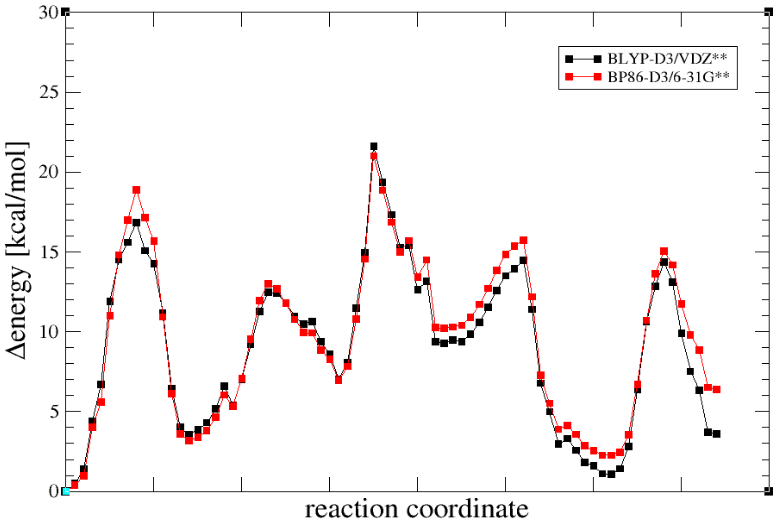

Fig. SI-2: Comparison of BP86-D3/6-31G** with BLYP-D3/VDZ** for the whole repair mechanism of FapydG by Fpg. The system consists of 54412 atoms in total and 87 atoms in the QM region.

SI-2 Discussion of different X-ray-structures of Fpg

As only one structure exists for FapydG, X-ray structures including 8OG need to be used for comparison. Two different structures for the educt state have been obtained via X-ray crystallography. One structure was obtained by a E2Q mutation [PDB-code: 1R2Y [1]] the other structure by replacing 8OG with the carbocyclic analogue c8OG [PDB-code: 4CIS [2]]. The three structures have in common, that they trap the educt state, but it is worth mentioning, that only the crystal structures, where the damaged nucleotide was substituted by a carbocyclic compound[2, 3], contain a water molecule in the active site (X-WAT).

The only intermediate structure published so far is a Schiff base intermediate, where 8OG has been cleaved and the damaged base could not be resolved in the binding pocket[4] [PDB-code: 1L1Z]. Therefore no information can be gained about the presence of the water molecule in this structure.

The X-ray structure of the DNA-enzyme complex containing cFapydG [PDB-code: 1XC8] discussed in more detail:

In contrast to FapydG, cFapydG cannot be cleaved by the enzyme. cFapydG blocks the reaction by lacking the $O_{4'}$ atom, which was replaced by a carbon atom. In addition, no hydrogen bonds can be formed anymore to the ribose. Interactions between Fpg and $O_{4'}$ have been disabled by the modification, hence, the interaction pattern in the active site is expected to have significantly changed and to be disturbed. This indicates, that the interaction between the active site (especially E2) and $O_{4'}$ is crucial for the reaction.

The presence of a water molecule in the active site in some of the structures raises the question whether it is part of the excision mechanism *in vivo* or only an artifact of the crystallization conditions.

SI-3 Details of the FF-MD simulations

SI-3.1 Behavior of different systems in FF-MD

We employed FF-MD, which is a standard method to investigate the overall dynamics of systems by gaining a huge number of snapshots. This method can be applied because no chemical reaction takes place. To gain deeper insights into the behavior of the active site, multiple FF-MD simulations for all four systems have been performed: FapydG with (I) and without X-WAT (II), cFapydG with (III) and without X-WAT (IV). For proper statistics and to obtain a statistically significant analysis, at least 5 simulations have been performed for each system. The average runtime over all systems was 110 ns. The simulation time for each system is listed in Tab. SI-1. For system III more simulations have been performed to have better insight into the behavior of X-WAT. A long simulation time was used to investigate if other water molecules again enter the active site of Fpg.

Tab. SI-1: FF-MD systems

system	Total time	Number of simulations
I (FapydG including X-WAT)	100 ns	5
II (FapydG without X-WAT)	70 ns	5
III (cFapydG including X-WAT)	150 ns	12
IV (cFapydG without X-WAT)	120 ns	5

In the FF-MD simulations of FapydG including X-WAT (system I), X-WAT inhibits the protonated E2 to form a hydrogen bond with $O_{4'}$ which is needed to allow the starting conformation for the reaction to occur (see Section "repair mechanism"). X-WAT changes the hydrogen network within the active site substantially: It can form H-bonds to E5 (acceptor), amino-group of FapyG (donor), E2 (donor), and $O_{4'}$ (acceptor). Hence, X-WAT prevents the interaction between $O_{4'}$ and E2 due to steric hindrance. E2 interacts with $O_{3'}$ instead. X-WAT can also leave the active site

and then stay in the solvent within the simulation time. In this case no other water molecule enters the active site.

Also during FF-MD simulations of FapydG without X-WAT (system II), no entrance of a water molecule into the active site has been observed, and there are multiple events of interaction between protonated E2 with $O_{4'}$ (see SI-2.3). We also performed FF-MD calculations on the DNA-enzyme complex containing cFapydG instead of FapydG. In that case, X-WAT leaves the active site within the first ns in most cases (system III). In system IV no X-WAT is present in the beginning of the simulations. For this system we have observed that a water molecule is able to enter the active site with low probability after about 30 ns.

SI-3.2 Structural analysis of FF-MD

As a stability check of the system during the FF-MD simulations, RMSD of the protein backbone, DNA and X-WAT are plotted in Fig. SI-3. As reference for the structure analysis we use the minimized and equilibrated X-ray structure. The selection of DNA consists of the backbone atoms of FapydG with pairing nucleotide as well as the previous and following nucleotide pair, where hydrogens were excluded.

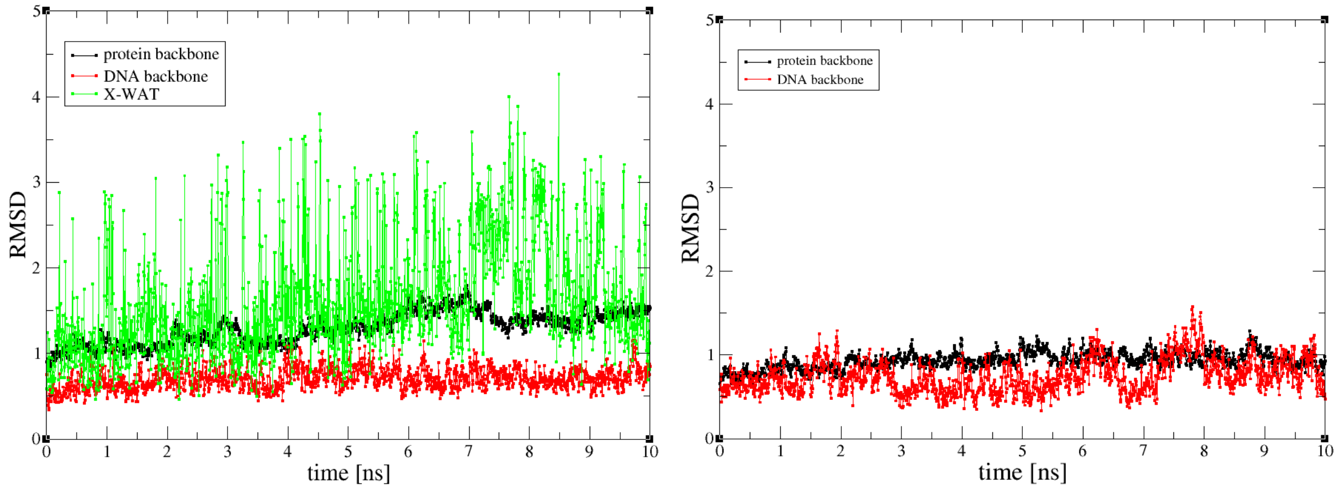

SI-Fig.3: RMSD plots for systems I (left) and II (right) of the protein backbone, DNA, and X-WAT. The system is more stable without X-WAT, due to the direct interactions between $O_{4'}$ and E2.

SI-3.3 Interaction between ribose and E2

Tab. SI-2 shows that the crucial interaction between $O_{4'}$ and E2 is much more probable without X-WAT. For definition of the systems see Tab. SI-1. Hydrogen bonding is defined as ≤ 3 Å between donor and acceptor with an angle cutoff of 45°.

Tab. SI-2: Interactions between ribose and E2 in system I + II.

Interaction between	donor/acceptor	occupancy system I	occupancy system II
$O_{4'}$ and E2	donor GLH2-side	0.48%	36.97%
	acceptor FapyG278-side		
$O_{3'}$ and E2	donor GLH2-side	73.81%	37.52%
	acceptor FapyG278-side		

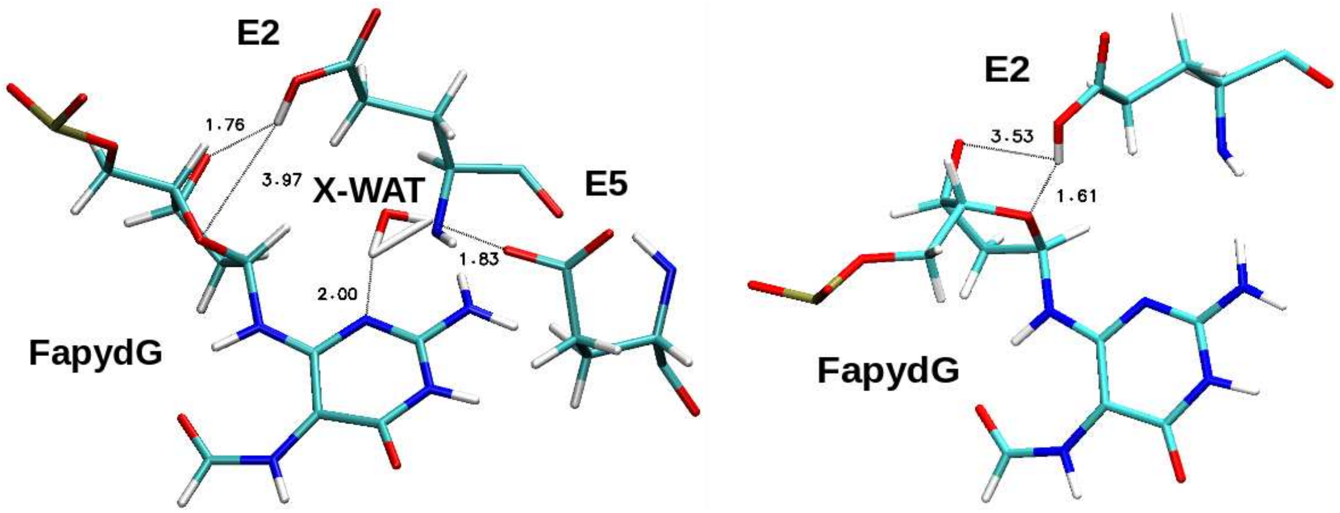

Fig. SI-4: Left: system I, where a snapshot out of a MD simulation is shown as example of the disturbed hydrogen network. The distance of E2 to $O_{4'}$ is much bigger than to $O_{3'}$ due to steric hindrance of X-WAT, which in this snapshot forms hydrogen bonds to N_3 of FapyG and to E5, both as hydrogen donor. Right: system II, where a snapshot out of a MD simulation is shown as example of the hydrogen network. E2 is much closer to $O_{4'}$ than to $O_{3'}$, so the system gets into a suitable starting conformation for the repair reaction.

SI-4 Details of the reaction mechanism

SI-4.1 Glycosidic bond breakage for all possible protonation states

For all eight possible protonation states (of P1, E2, E5) calculations of the direct glycosidic bond breakage have been performed. For each protonation state these calculations have been performed for the system containing X-WAT and lacking X-WAT. The nomenclature of the protonation states is listed in Tab. SI-3. The reaction profiles are depicted in Fig. SI-5.

Tab. SI-3: Overview of all eight possible protonation states in the active site of Fpg.

	P1	E2	E5
I	−	−	−
II	−	−	+
III	+	−	+
IV	+	−	−
V	−	+	−
VI	+	+	−
VII	−	+	+
VIII	+	+	+

(+) protonated; (-) unprotonated

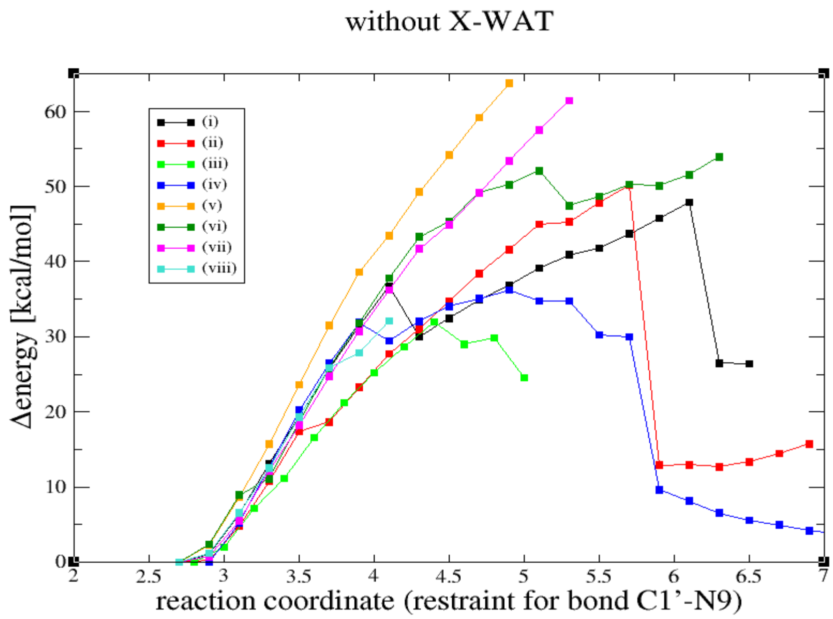

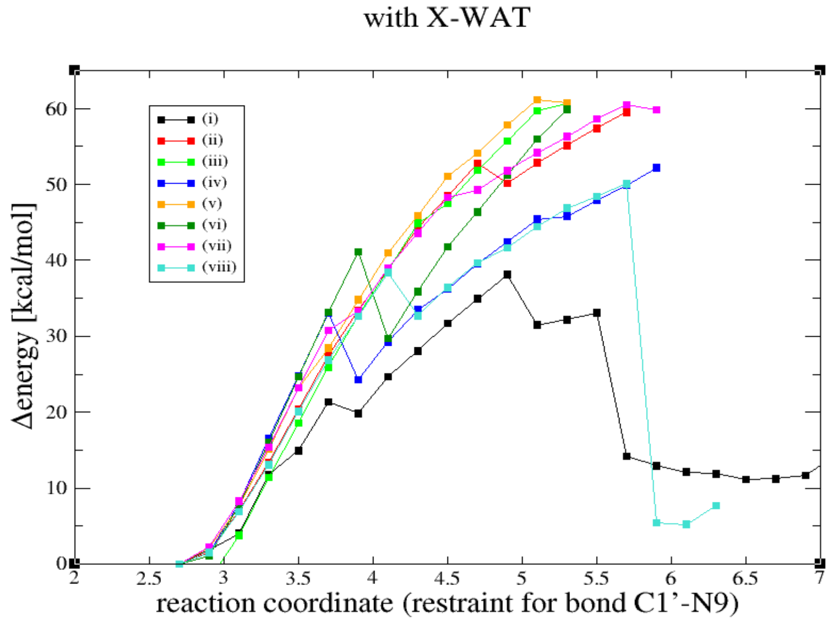

Fig. SI-5: Simulation of direct glycosidic bond breakage for all eight possible protonation states - without X-WAT and with X-WAT, respectively. No reaction barrier is lower than 30 kcal/mol. The system for these calculations consists of the whole equilibrated structure (54412 atoms), with 10 Å around N_9 of FapydG as relaxed region and about 90 QM atoms, depending on the protonation state and presence of X-WAT.

SI-4.2 Structure of Schiff base intermediate

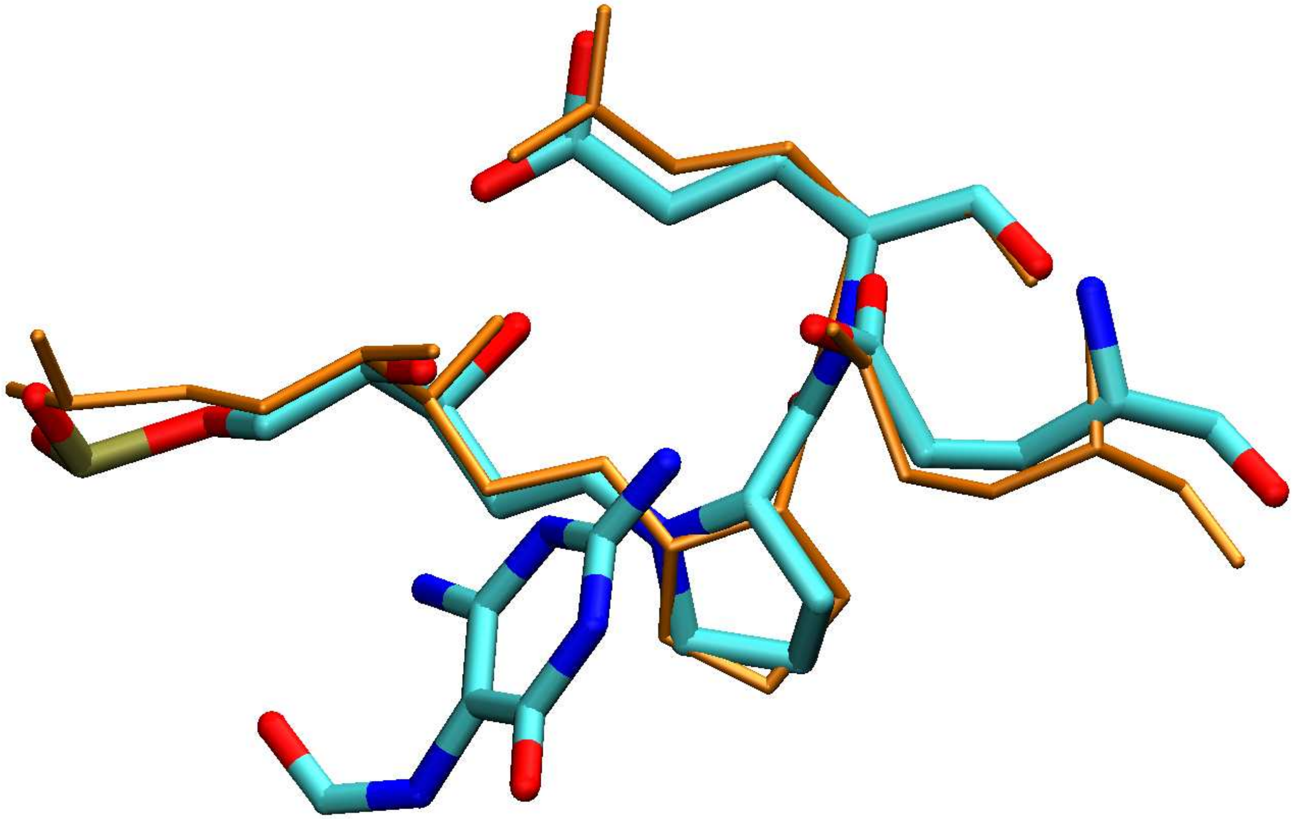

Fig. SI-6: Alignment of the obtained QM/MM calculated Schiff base and the corresponding X-ray structure [PDB-code: 1L1Z]. The calculated structure is shown in atomic colors, the structure obtained by X-ray crystallography is shown in orange. In the X-ray structure the cleaved base is not resolved.

SI-5 Details on the QM size convergence (QM/MM)

SI-5.1 Selected residues for each QM sphere within the QM size convergence study

Tab. SI-4: selection of residues included in QM/MM calculations for QM size convergence

QM atoms	Residues of QM atoms	Number of residues (QM)	Atoms in relaxed region	Residues of relaxed region	Number of residues in relaxed region	Charge (QM)
87	1 2 5 278	4	754	1-6, 57, 72-78, 109, 111, 161, 170-173, 216-223, 232, 235, 238, 260, 277-279, waters: 312 314 315 336 347 353 359 360 362 372 375 395 412 419 440 461 479 488 498 874 1295 1333 1337 1339 1344 1346 1498 1506 1522 1865 1888	67	-2
218	1 2 5 75 172 219 222 277 278 waters: 336 461 498 1337 1506	14	754	1-6, 57, 72-78, 109, 111, 161, 170-173, 216-223, 232, 235, 238, 260, 277-279, waters: 312 314 315 336 347 353 359 360 362 372 375 395 412 419 440 461 479 488 498 874 1295 1333 1337 1339 1344 1346 1498 1506 1522 1865 1888	67	-3
515	1 2 5 57 74 75 76 109 170 171 172 173 217 218 219 220 221 222 238 260 277 278 279 waters: 314 315 336 359 375 440 461 498 1337 1339 1344 1498 1506 1865	37	218	1 2 5 75 172 219 222 277 278 waters: 336 461 498 1337 1506	14	0
622	1 2 5 6 57 73 74 75 76 77 109 170 171 172 173 217 218 219 220 221 222 223 235 238 260 277 278 279 waters: 312 314 315 336 359 362 372 375 395 419 440 461 479 488 498 874 1333 1337 1339 1344 1498 1506 1522 1865 1888	53	218	1 2 5 75 172 219 222 277 278 waters: 336 461 498 1337 1506	14	0
700	1 2 4 5 6 57 72 73 74 75 76 77 78 109 170 171 172 173 217 218 219 220 221 222 223 232 235 238 260 277 278 279 waters: 312 314 315 336 359 360 362 372 375 395 412 419 440 461 479 488 498 874 1333 1337 1339 1344 1498 1506 1522 1865 1888	59	218	1 2 5 75 172 219 222 277 278 waters: 336 461 498 1337 1506	14	1

SI-5.2 QM size convergence study of the reaction mechanism

Tab. SI-4: QM size convergence study of the reaction mechanism. Values in kcal/mol relative to the respective educt.

QM 87	QM 218	QM 515	QM 622	QM 700	comment
0	0	0	0	0	educt
17.7	12.0	10.3	10.8	10.5	
19.1	9.4	11.9	12.9	13.0	
20.5	8.8	11.6	14.2	14.1	1st barrier
19.9	7.1	9.2	10.7	11.0	
7.6	-7.7	-4.0	-1.4[a)]	-1.3[a)]	1st intermediate
16.7	1.8	0.9	7.5[a)]	7.6[a)]	2nd barrier
16.5	6.0	2.4	4.1	4.3	
14.7	2.2	1.3	4.7	4.5	
12.0	-0.7	-0.2	3.0	3.6	2nd intermediate
18.7	11.1	10.4	14.8	14.4	
24.9	23.0	17.4	20.1	19.9	
22.8	23.4	18.6	21.6	21.1	3rd barrier
20.9	21.0	16.1	19.3	18.9	
15.1	9.8	13.2	17.2	16.9	3rd intermediate
15.6	11.5	13.8	18.1	16.8	
16.1	15.7	14.8	17.7	17.2	
19.0	14.4	16.8	21.0	19.6	4th barrier
7.8	9.7	9.3	12.4	11.0	
7.3	5.7	5.9	8.9	8.5	4th intermediate
19.0	9.9	14.5	18.8	17.5	5th barrier
19.3	17.7	15.2	17.9	16.8	
19.0	6.1	10.5	13.2	12.5	
11.8	-4.5	-1.5	1.9	2.5	product

[a)] Since the SCF cycle did not converge for BP86-D3/6-31G** calculations due to the small HOMO-LUMO gap, we estimated this value via B3LYP-D3/6-31G** calculations (for details see SI-5.3).

SI-5.3 Discussion of estimated energies in the QM size convergence (QM/MM)

When we converged the relative energies for the larger systems of the barriers and intermediate structures, we encountered severe convergence problems for the 1st intermediate and one structure of the 2nd barrier calculated with BP86-D3. The problem of vanishing HOMO-LUMO gaps in DFT calculations for large systems is well known[5, 6]. To estimate the energy of these two structures, we choose B3LYP-D3, since the calculated HOMO-LUMO gap is larger for hybrid functionals, due to the exact HF exchange, allowing convergence of the SCF calculation.

We calculated single point (sp) energies with B3LYP-D3/6-31G** on the structures, where the active site (218 QM atoms) has been optimized with BP86-D3/6-31G**, and increased the QM region stepwise. Tab. SI-6 contains sp energies for all barriers and intermediates, calculated with B3LYP-D3/6-31G**. Compared to the energies calculated with BP86, the B3LYP values are higher in energy, but the convergence behavior is almost the same. In Fig. SI-7 two examples are shown. Since the convergence behavior and the average increase of ΔE from QM218 to QM700 show the same behavior for BP86-D3 and B3LYP-D3 (examples shown in Fig. SI-7), we used the increase of the B3LYP-D3 energies to estimate the missing energies of the BP86-D3 calculations. The average ratio of B3LYP-D3(700QM-218QM) to BP86-D3(700QM-218QM) is 1.2. We estimate the missing BP86-D3 energies by dividing the increase of B3LYP-D3 by this factor to get estimated the increase of BP86-D3 and add it to the QM218 energy of BP86-D3.

Based on this estimation, we conclude, that the exact energies of the two structures would not change the overall picture of our reaction profile. It should be noted, that although in general sp calculations are enough to estimate the necessary size of the QM region, that has to be included to obtain size converged energies, geometry optimizations are necessary to calculate the influence of this QM region on the energy.

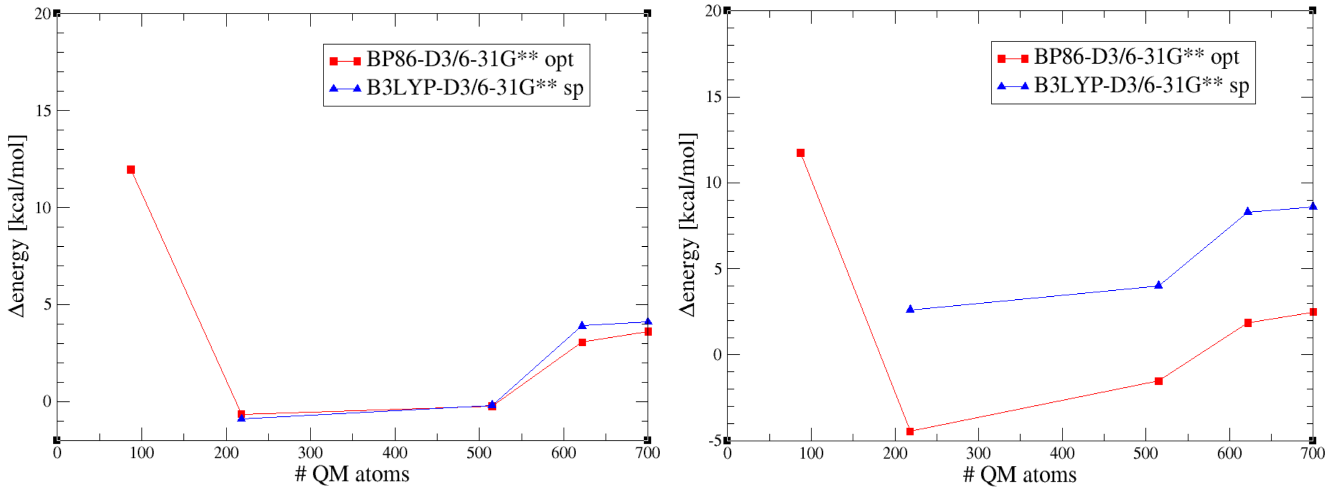

Fig. SI-7: Relative energies are plotted against the QM size within the QM/MM calculations for two different reaction points: 2nd intermediate (left), product (right) calculated with BP86-D3/6-31G** (opt) and B3LYP-D3/6-31G** (sp energy).

Tab. SI-6: QM size convergence study of the reaction mechanism, where the crucial points of the reaction have been calculated with increasing QM region in sp calculations (see text) with B3LYP-D3/6-31G**. All values are in kcal/mol relative to the respective educt.

218	515	622	700	comment
0	0	0	0	educt
15.7	13.7	14.9	14.8	
13.4	17.3	19.5	19.4	
13.7	17.6	20.6	21.2	1st barrier
12.7	15.3	18.6	18.8	
-3.1	0.8	4.5	4.6	1st intermediate
4.8	7.5	11.6	11.7	
8.8	7.5	10.6	10.8	
5.6	7.2	11.4	11.5	2nd barrier
2.6	4.0	8.3	8.6	2nd intermediate
15.1	14.7	20.1	20.2	
26.9	20.9	24.7	24.6	
25.9	20.9	24.9	24.0	3rd barrier
23.3	18.5	22.5	21.7	
11.8	15.7	20.4	19.6	3rd intermediate
13.5	16.4	21.0	20.2	
17.6	18.9	22.4	21.6	
16.5	19.5	23.9	23.1	4th barrier
10.6	9.5	12.9	12.3	
7.3	9.2	12.9	12.2	4th intermediate
18.0	19.5	24.1	23.5	5th barrier
25.8	21.5	24.0	23.3	
13.8	15.8	19.7	19.3	
-0.9	-0.2	3.9	4.1	product

SI-6 Detailed Methods Section

SI-6.1 Details for Molecular Dynamics Simulations

The following steps have been performed: The systems were energy minimized (NVT ensemble) in 3 steps, relaxing different degrees of freedom, using of the conjugate gradient algorithm: (1) only hydrogen atoms (2000 steps), (2) only solvent (3000 steps), (3) all atoms (5000 steps). The system was heated up to 300 K within 10 ps using Langevin dynamics, where a positional constraint of 1 kcal/mol/$Å^2$ was applied on non-water atoms. In the subsequent equilibration step we switched to the NPT ensemble employing the Langevin piston Nosé-Hoover method [7, 8]. At this stage the restraints on non-water atoms are reduced step by step down to zero (0.2 kcal/mol/$Å^2$ increments for every 20 ps). Equilibration was then performed for 300 ps, in which the coordinates were saved every 0.2 ps. Production runs were performed for at least 10 ns with timesteps of 2 fs using the SHAKE algorithm [9]. Coordinates were saved every 6 ps.

SI-6.2 Details for QM/MM calculations

The system for these calculations consists of the whole equilibrated structure (54412 atoms), with 15 Å around N_9 of FapydG as relaxed region and 87 QM atoms. For the QM size convergence study the total system was reduced to protein, DNA, ions, water within 15Å of N_9 of FapydG, and 3 Å of water around both protein and DNA (in total 12031 atoms in the system).

References

1. Fromme, J. C. & Verdine, G. L. DNA lesion recognition by the bacterial repair enzyme MutM. *J. Biol. Chem.* **278,** 51543–8 (2003).

2. Sadeghian, K. *et al.* Ribose-protonated DNA base-excision repair: a combined theoretical and experimental study. *Angew. Chem., Int. Ed. doi: 10.1002/anie.201403334R1* (2014).

3. Coste, F. *et al.* Structural basis for the recognition of the FapydG lesion (2,6-diamino-4-hydroxy-5-formamidopyrimidine) by formamidopyrimidine-DNA glycosylase. *J. Biol. Chem.* **279,** 44074–44083 (2004).

4. Fromme, J. C. & Verdine, G. L. Structural insights into lesion recognition and repair by the bacterial 8-oxoguanine DNA glycosylase MutM. *Nat. Struct. Biol.* **9,** 544–52 (2002).

5. Rudberg, E., Rubensson, E. H. & Salek, P. Kohn–Sham Density Functional Theory Electronic Structure Calculations with Linearly Scaling Computational Time and Memory Usage. *J. Chem. Theory Comput.* **7,** 340–350 (2011).

6. Lever, G., Cole, D. J., Hine, N. D. M., Haynes, P. D. & Payne, M. C. Electrostatic considerations affecting the calculated HOMO-LUMO gap in protein molecules. *J. Phys.: Condens. Matter* **25,** 152101 (2013).

7. Nosé, S. A unified formulation of the constant temperature molecular dynamics methods. *J. Chem. Phys.* **81,** 511–519 (1984).

8. Hoover, W. G. Canonical dynamics: Equilibrium phase-space distributions. *Phys. Rev. A* **31,** 1695–1697 (3 1985).

9. Ryckaert, J.-P., Ciccotti, G. & Berendsen, H. J. C. Numerical integration of the cartesian equations of motion of a system with constraints: molecular dynamics of n-alkanes. *J. Comp. Phys.* **23,** 327 –341 (1977).
